# Global Analysis Reveals Families of Chemical Motifs Enriched for hERG Inhibitors

**DOI:** 10.1371/journal.pone.0118324

**Published:** 2015-02-20

**Authors:** Fang Du, Joseph J. Babcock, Haibo Yu, Beiyan Zou, Min Li

**Affiliations:** The Solomon H. Snyder Department of Neuroscience, High Throughput Biology Center and Johns Hopkins Ion Channel Center (JHICC), Johns Hopkins University, 733 North Broadway, Baltimore, MD 21205, United States of America; Oak Ridge National Laboratory, UNITED STATES

## Abstract

Promiscuous inhibition of the human *ether-à-go-go*-related gene (hERG) potassium channel by drugs poses a major risk for life threatening arrhythmia and costly drug withdrawals. Current knowledge of this phenomenon is derived from a limited number of known drugs and tool compounds. However, in a diverse, naïve chemical library, it remains unclear which and to what degree chemical motifs or scaffolds might be enriched for hERG inhibition. Here we report electrophysiology measurements of hERG inhibition and computational analyses of >300,000 diverse small molecules. We identify chemical ‘communities’ with high hERG liability, containing both canonical scaffolds and structurally distinctive molecules. These data enable the development of more effective classifiers to computationally assess hERG risk. The resultant predictive models now accurately classify naïve compound libraries for tendency of hERG inhibition. Together these results provide a more complete reference map of characteristic chemical motifs for hERG liability and advance a systematic approach to rank chemical collections for cardiotoxicity risk.

## Introduction

Potassium currents conducted by the human *ether-à-go-g*o-related gene (hERG) channel re-polarize the membrane during cardiac contraction [1]. Reduction of hERG current density by unintentional drug block or genetic mutations often slows this repolarization and thereby prolongs the action potential. Because this prolongation increases the QT interval (the time period between de- and repolarization of the ventricular muscles during heartbeat) measured in surface electrocardiogram (ECG), it is commonly termed long QT syndrome (LQTS) [2], which poses significant risk for life-threatening arrhythmias. Drugs of diverse chemical structures have been withdrawn from the market due to this unintended inhibition [3]. Consequently, investigating the hERG effect of candidate drugs has become a critical part of safety assessment.

The hERG inhibition by known drugs and a limited number of drug-like compounds has been acquired by different experimental methods and previously annotated [4]; these structures represent many distinct chemotypes [5]. Such data have provided opportunities to develop *in silico* methods for predicting hERG liability by taking advantage of shared chemical patterns [4,6–11]. However, such methods have displayed inconsistent performance in *de novo* prediction. One explanation for such inconsistent predictability is that many hERG-inhibitory chemical motifs, especially compounds in naïve chemical libraries (libraries that are developed through diversity synthesis rather than targeted at previously explored drug-like space), are not represented by existing data. Larger datasets with greater coverage of previously unexplored chemical space may therefore be required to assemble a catalog of such features and improve performance [10,12]. Another potential contributing factor for the inconsistency relates to uniformity of existing data since inhibition profiles from different experimental methodologies, despite high quality, are not always comparable. For example, patch clamp measurements are the gold standard to assess channel activity. Data derived from a single high-quality methodology, e.g., electrophysiology, would therefore avoid discrepancies that may arise among different assay technologies previously used to assess hERG blockade [13–15]. Thus, we hypothesized that improved classifiers of hERG inhibition may be achievable by acquiring high-resolution electrophysiology measurements and by covering an expansive chemical library.

Among several major commercial chemical libraries, the National Institutes of Health (NIH) Molecular Library Small Molecule Repository (MLSMR) contains more than 300,000 structurally diverse compounds and as of 2012 this collection has been screened against 5000 peer-review selected protein targets [16]. We reasoned that, in addition to the intended purpose discussed above, the results will be valuable to prioritize active compounds in other screens. Inspired by analyses of social communities [17], protein interactions [18], and other complex systems [19], we constructed a network of compound ‘nodes’ overlaid with their hERG activity profiles. We then systematically explored communities, by asking whether compounds with differing hERG liability form distinct structural clusters, which may represent filters to develop more effective classifiers defining high-risk neighborhoods in naïve chemical space.

## Results

### High-throughput screen for chemical inhibition of hERG

To survey the chemical landscape of small molecule-mediated hERG inhibition, we performed electrophysiological measurements of hERG activity at 1 and 10 μM for each compound in the MLSMR collection. This collection contains both known bioactives, natural products, commercial compound collections, and a large percentage (>90%) of ‘diversity products’ derived from combinatorial chemistry that are intended to enrich regions of structural space not covered by well-characterized compounds [13] (see **Methods** for assay details). The quality of the data is validated by several performance statistics and experimental confirmation. Among the tested compounds, 306,985 (>96%) passed quality control (QC) filters and were annotated for percent inhibition based on level of inhibition of tail currents before and after compound treatments. Compounds which failed in QC include those disrupting cell membranes and those assayed in defective wells in patch plates. The latter resulted from insufficient seal resistance in either individual wells or whole plates.

### Structural neighborhoods of hERG inhibitors

**Fig 1:**
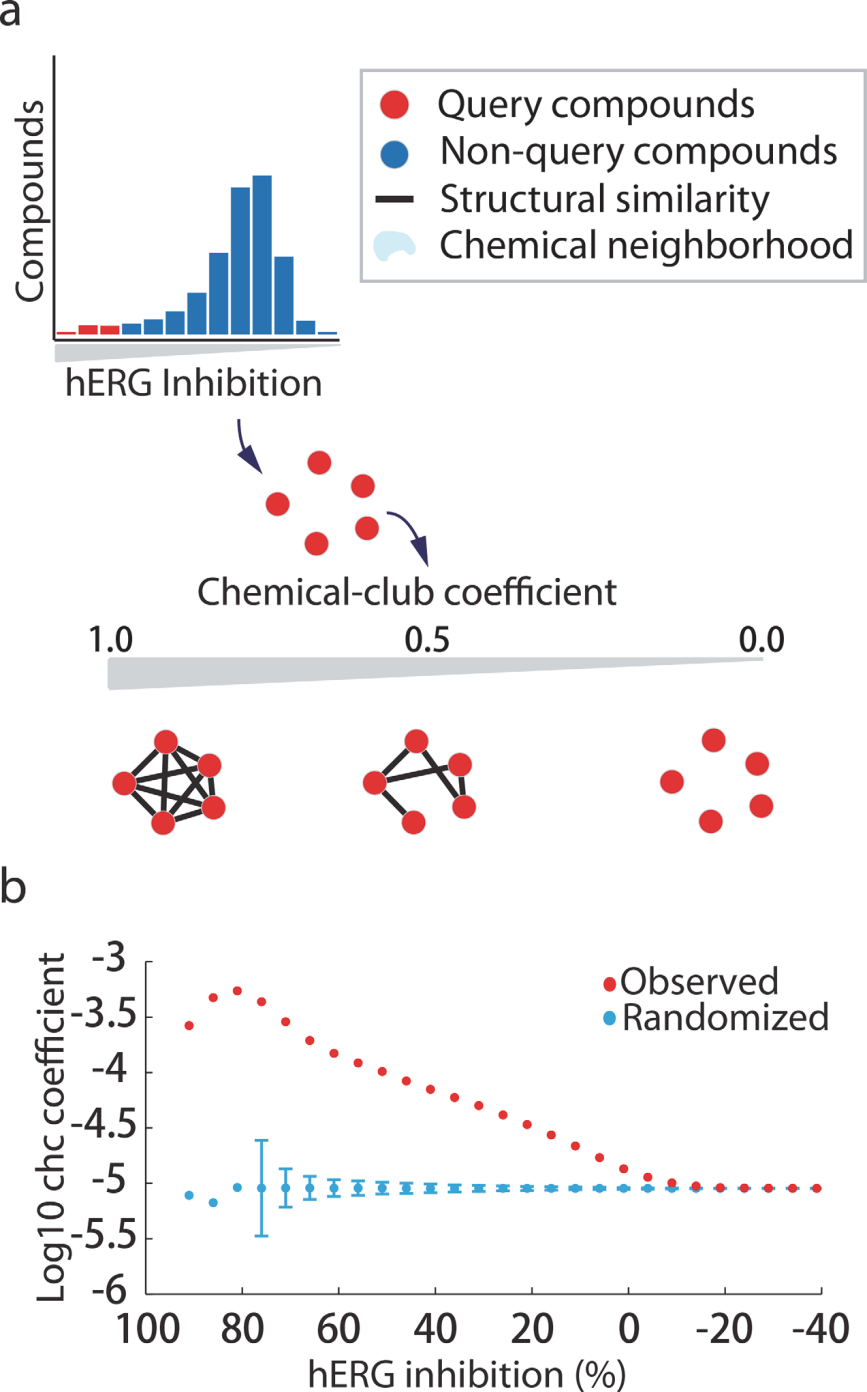
Potent hERG blockers exhibit preferential structural similarity. (**a**) Schematic diagram of chemical structure network analysis. The chemical-club coefficient (ChC) measures the density of connections (structurally similar pairs) among query compounds (red nodes) above a given threshold of hERG inhibition (top, red bars). (**b**) ChC calculation plotted for the MLSMR library for randomized (blue, mean +/- 3 standard deviations for randomized datasets) and observed (red) activity for 10 μM data, where compound adjacency is judged by a Tanimoto Coefficient > 0.7 for FCFP_6 circular fingerprints.

Similar to what has been proposed by others [20–26], we hypothesized that hERG blockers identified by our screen may share certain structural features correlated with their inhibitory profile, and thus occupy nearby regions of chemical space. Differently from the earlier studies, our dataset is considerably larger and acquired by one methodology. To explore this idea, we organized the MLSMR library in a network where nodes represent compounds linked by edges if they share structural similarity using multiple algorithms including 2D chemical fingerprints (denoted 2D), overlap of 3D conformations (denoted 3D), and hierarchical relationships between scaffolds (denoted Scaffolds) defined by the Murcko algorithm [27–30]. We then systematically compared the structural neighborhoods of compounds in different ranges of hERG activity (i.e., inhibition) by computing the rich-club coefficient, a parameter previously utilized to quantify the tendency of nodes with many links to be very well connected to each other [31,32]. Because our calculation is based on an activity threshold instead of the more conventional node degree threshold, we term it the ‘chemical-club coefficient’ (ChC). The ChC ranges from 0 to 1, with higher values indicating greater density of structural similarity links among a set of compounds (**Fig. 1A**). For example, 10e-5 indicates the ratio of observed edges to the maximum number of possible edges between compounds (see **Methods**). The 2D ChC profile reveals higher than expected similarity among potent hERG inhibitors compared to a randomized baseline, quantified statistically by lack of enhanced ChC among potent inhibitors in 1,000 randomized sets (empirical p-value <0.001, see **Methods**) (**Fig. 1B**). While the observed and randomized density of structurally similar pairs between potent hERG inhibitors differs by two orders of magnitude, the observed density is still below the maximum of ChC = 1 (i.e., if all inhibitors shared structural similarity) suggesting that these compounds occupy several distinct structural neighborhoods instead of aggregating in a single giant community. While the observed ChC values do not directly indicate a number of communities, upper bound calculations are given in **Methods**. The generality of the above statistics is indicated by similar results obtained when edges in our network are defined using two alternative structural similarity criteria (Scaffold or 3D), with more potent compounds or scaffolds displaying statistically significant peaks in the ChC profile (**S1A,B Fig. respectively**). For the Scaffold network, the ChC profile achieves the same peak, but declines more rapidly with compound potency (**S1A Fig.**). For 3D, the peak is significantly reduced in magnitude (**S1B Fig.**). Furthermore, the merging of the 2D and 3D similarity criteria in the ChC calculation reduces the gap between the randomized and empirical potent inhibitor peak in **Fig. 1B**, suggesting that simple 2D molecular geometry best partitions hERG inhibitors from inactive chemical space (**S1C Fig.**) (note that the Scaffold network cannot be merged with 2D, 3D because it is a fragment-fragment, not compound-compound network). This may be explained by the 3D set not necessarily containing the biologically active conformer of a compound, and thus similarity pairs may be based on inactive-inactive comparisons which dilute the correlation between biological and chemical similarity. Concordant observations were made in a comprehensive analysis of the MLSMR that compared screening hits to inactives across many biological targets [33]. Further, the observed 2D ChC profile is robust to exclusion of known drugs and bioactives in the MLSMR collection, indicating that they represent potentially novel hERG inhibitory chemotypes (**S1D Fig.**). Importantly, the number of structural neighbors of a compound is not itself strongly associated with hERG inhibition (Pearson correlations of -0.053, 0.003, and 0.0725, respectively, for the three similarity metrics), suggesting these observations cannot be explained only by the frequency of particular scaffolds in our dataset. Taken together, these analyses reveal that potent hERG inhibitors are proximal to each other under multiple definitions of structural similarity, and share a greater than expected density of connections distributed in multiple clusters in our structure network.

To compare these findings to the current chemical landscape of hERG inhibitors represented by publically available data, we chose two recently described collections containing 2,644 [4] and 368 [10] compounds assembled from literature sources, denoted D2644 and D368 (**S1 Table**). We selected these datasets based on the criteria that a) they had been used to develop models with predictive power in out-of-sample evaluation which could be re-implemented and b) they contain activity from diverse experimental sources, allowing us to evaluate the effect of such heterogeneity and c) they were the largest publically available datasets at the time of our analysis. The MLSMR library features a large percentage of ‘diversity’ compounds synthesized to probe regions of chemical space not represented by existing drugs [13]. Conversely, D2644 contains many known blockers and FDA-approved drugs, though these constitute 1,609 distinct murcko scaffolds and so are relatively diverse compared to each other [4]. While the D2644 data contains experimental measurements from electrophysiology and binding assays, as well as both mammalian and Chinese Hamster Ovary (CHO) cell systems, the D368 data was curated to include only electrophysiological data from mammalian systems, though still derived from multiple platforms (Ionworks [34], PatchXPress [35], and QPatch [36]) as well as manual recordings. Thus we could compare the effects of heterogeneity among multiple inhibition assays and variations of a single methodology (electrophysiology) on modeling results. These datasets may both be browsed on our website [37].

**Fig 2:**
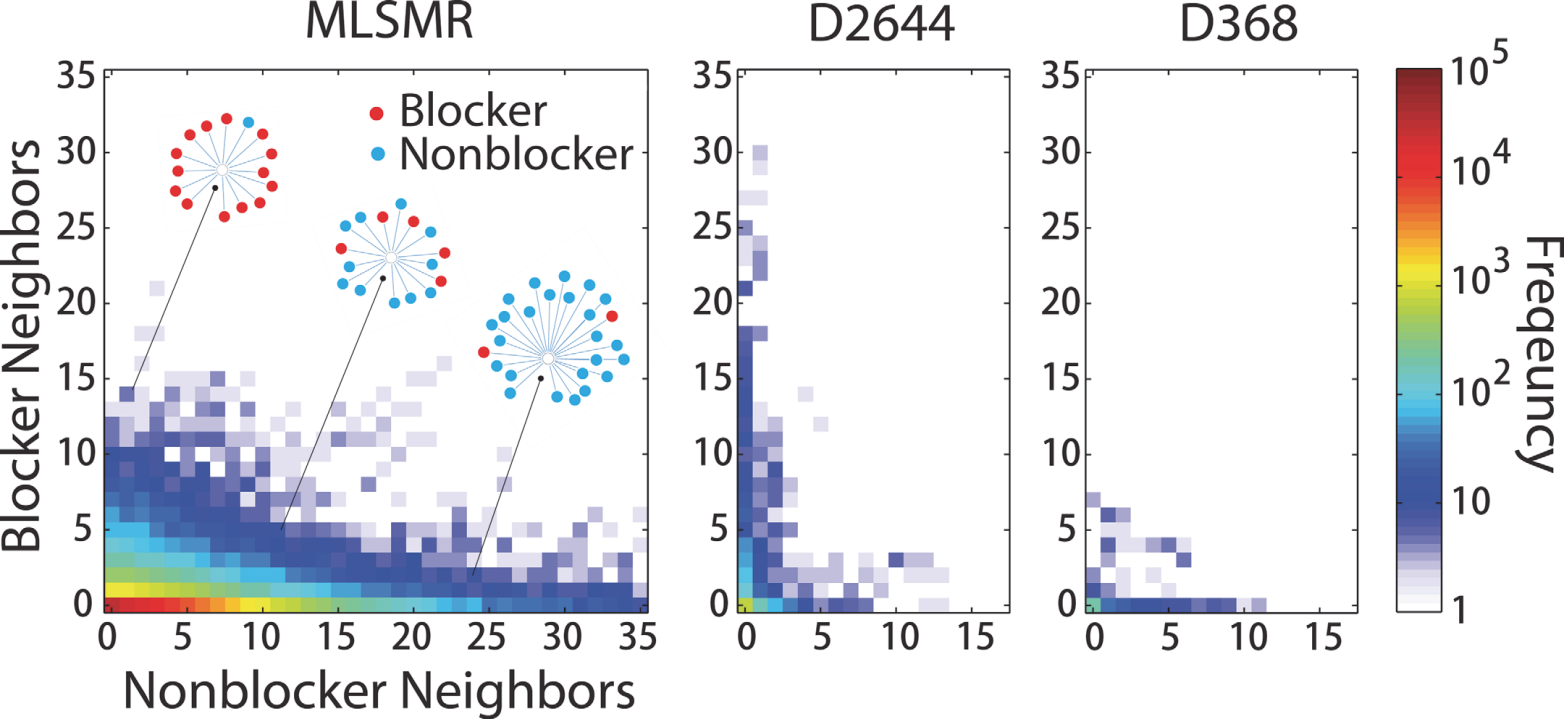
Continuity of structure variants between blockers and nonblockers in the MLSMR and previous hERG datasets. Each dataset is represented by a structure network as described in **Fig. 1**. Compound neighbors are classified as blocker (>50% hERG Inhibition at 10 μM) and nonblocker (<50%). The frequency of compounds with a given number of blocker and nonblocker neighbors in each dataset is plotted, with white cells representing empty data, and the origin representing singleton compounds with no neighbors. Grid points along the vertical axis (horizontal axis) represent compounds with a majority blocker neighbors (nonblocker neighbors). The region along the diagonal represents the transition zone where compounds possess mixed blocker and nonblocker neighbors. This transition zone is illustrated by three example neighborhoods containing blockers (red nodes) and nonblockers (light blue nodes).

Because the hERG actives in the D2644 and D368 sets are derived from different assays that may result in discordant continuous inhibition values for a single compound, these studies minimized this heterogeneity by constructing classification models from these data that utilize binary labels (blocker or nonblocker). Thus, for comparison, we also binarized the activity measurements in our data (with blockers >50% inhibition) and compared the distribution of chemical neighborhood phenotypes in the three collections using the same 2D network described in Fig. 1. The resulting grid plots the count of compounds in each collection with a given number of blocker and nonblocker neighbors (Fig. 2). Compounds with neighbors of predominantly one class are distributed along either the vertical or horizontal axis for all three datasets (Fig. 2), with the increased frequency of high-blocker neighborhoods in D2644 indicating duplicate data points for well-studied hERG inhibitors (**Fig. 2, center**). As D368 is more imbalanced between classes than D2644, the greater frequency of nonblockers to blockers is reflected in greater skew towards nonblocker neighbors along the horizontal axis (**Fig. 2, right**). The relative scarcity of blockers in our data is also reflected by the high density of compounds with nonblocker neighborhoods along the horizontal axis of the MLSMR plot. However, the transition zone of compounds possessing a mixture of blocker and nonblocker neighbors is most pronounced in the MLSMR (**Fig. 2, left**) but essentially missing in the other two datasets (**Fig. 2, center, right**). This observation correlates with the fact that many records in D2644 and D368 represent duplicate measurements of known hERG blockers, while the MLSMR contains previously uncharacterized blockers with many active and inactive derivatives generated through combinatorial chemistry. Other physiochemical parameters including molecular weight, ALogP, and polar surface area (PSA) also indicate greater diversity for the MLSMR collection (**S2 Fig.**). Thus, our analyses also highlight a richer distribution of neighborhood phenotypes in our large dataset than is currently represented by publically available collections.

### Ensemble modeling of drug-induced hERG inhibition

**Fig 3:**
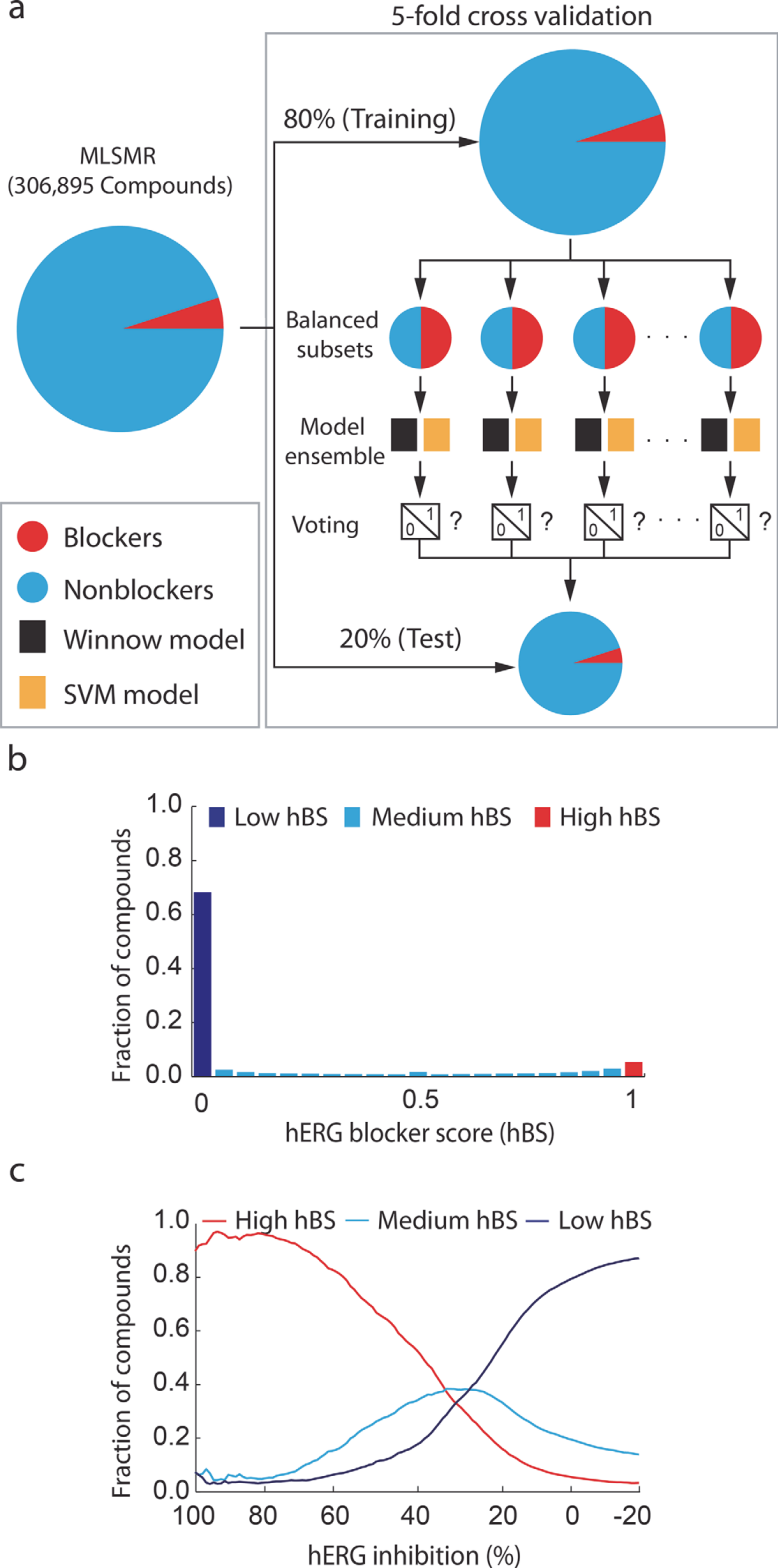
Ensemble modeling segregates compound populations by hERG liability. (**a**) Schematic of ensemble voting procedure. MLSMR compounds (left) are divided in 5 test folds (bottom), each receiving binary votes from an ensemble of hERG classifiers (center right) developed using balanced batches of the 80% training set not included in each test fold (top right). (**b**) The average vote of hERG blocker over all models in the ensemble is recorded as a hERG Blocker Score (hBS). Compounds with high hBS (red histogram bar) have consistent blocker classification, while those with low hBS (dark blue histogram bar) have consistent nonblocker classification. Compounds with intermediate hBS score are denoted by light blue histogram bars. (**c**) Distribution of hERG inhibition (10 μM) for compounds with High (>0.95), Intermediate (0.05<hBS<0.95) and Low (<0.05) hBS.

While the predictive classifiers developed using the D2644 and D368 sets exhibit excellent cross-validated predictions, considerable variation in performance was noted for independent, external data [4,10]. We also found reduced performance applying these models to our data (see **S3 Fig., S2 Table**), and hypothesized that re-training the algorithms using our screening results might better capture the neighborhood patterns described above. To evaluate this notion, we randomly divided the MLSMR into five folds and utilized a cross-validation procedure: in each round, four folds were used as training data and one as an independent test set. Like a typical naïve screening library, a small fraction of the MLSMR compounds are hERG blockers (approximately 5%). To avoid class-specific bias toward the majority class (inactive compounds) during model optimization we randomly generated balanced subsets of the training data and used these to generate an ensemble of models from the D2644 and D368 algorithms [38] (Fig. 3A). The individual models in the ensemble yielded predictions (votes) of blocker (1) or nonblocker (0) for each compound in the test set (Fig. 3A). Analysis of individual and combined performance of the models indicated that averaging the results of both yielded better predictions (**S4 Fig.**). In addition, the ensemble strategy used here can output a quantitative score to rank compounds in terms of their likeliness of being blockers. This allows for evaluating the predictive model with more rigorous analysis including receiver operating characteristic (ROC), which is not available in the original models where the outputs are 0/1 class labels. Specifically, the average vote was calculated as a hERG Blocker Score (hBS) ranging from 0 to 1, with higher values indicating consistent votes for blocker (**Fig. 3B**). While more than half the library received hBS values near 0, a large fraction also received intermediate votes, indicating variable predictions dependent upon the particular training subsets used to generate members of our model ensemble (**Fig. 3B**). A distinct population of approximately 5% of compounds received consistent blocker votes, a pattern similar to the potent neighborhoods described in Fig. 1. The resulting distribution of hERG inhibition for compounds in three ranges of hBS (*i*.*e*., high >0.95, intermediate between 0.95 and 0.05, or low <0.05) demonstrates correct segregation of compound populations with respect to their continuous hERG inhibition measurements (**Fig. 3C**). Our results also demonstrate reasonable classification of the D368 and D2644 data using this retrained models, with higher MCC than the original models applied to the MLSMR (**S3C,F Fig., S2 Table**). The neighborhood diversity of moderate inhibitors is suggested by the large fraction of these compounds with intermediate hBS scores, reflecting variable classification dependent upon a particular ensemble member’s training subset (**Fig. 3C**). Potent inhibition correlates with high hBS, an intriguing result because the binary classifiers in the ensemble do not incorporate the magnitude of inhibition above or below the 50% threshold (**Fig. 3C**). Furthermore, this pattern suggests that the neighborhoods of potent hERG blockers revealed by our network analysis are readily identified by *in silico* methods.

### Network patterns of *in silico* predictions

We next investigated how compounds with *in silico* classifications of varying accuracy are distributed in the structure network described in **Fig. 1**, using the distribution of hBS scores and annotated activities to divide the MLSMR into three major classes based on predictability: those that are correctly predicted (either as blocker or nonblocker) by most models in our ensemble, those that are misclassified by most models, and those with inconsistent votes (represented by intermediate hBS scores). We labeled compounds in these three groups as predicable (P), unpredictable (U), or inconsistent (I). Combined with our earlier annotation of each compound as blocker (B) or nonblocker (NB), this process yields six activity-predictability classes for the MLSMR data.

**Fig 4:**
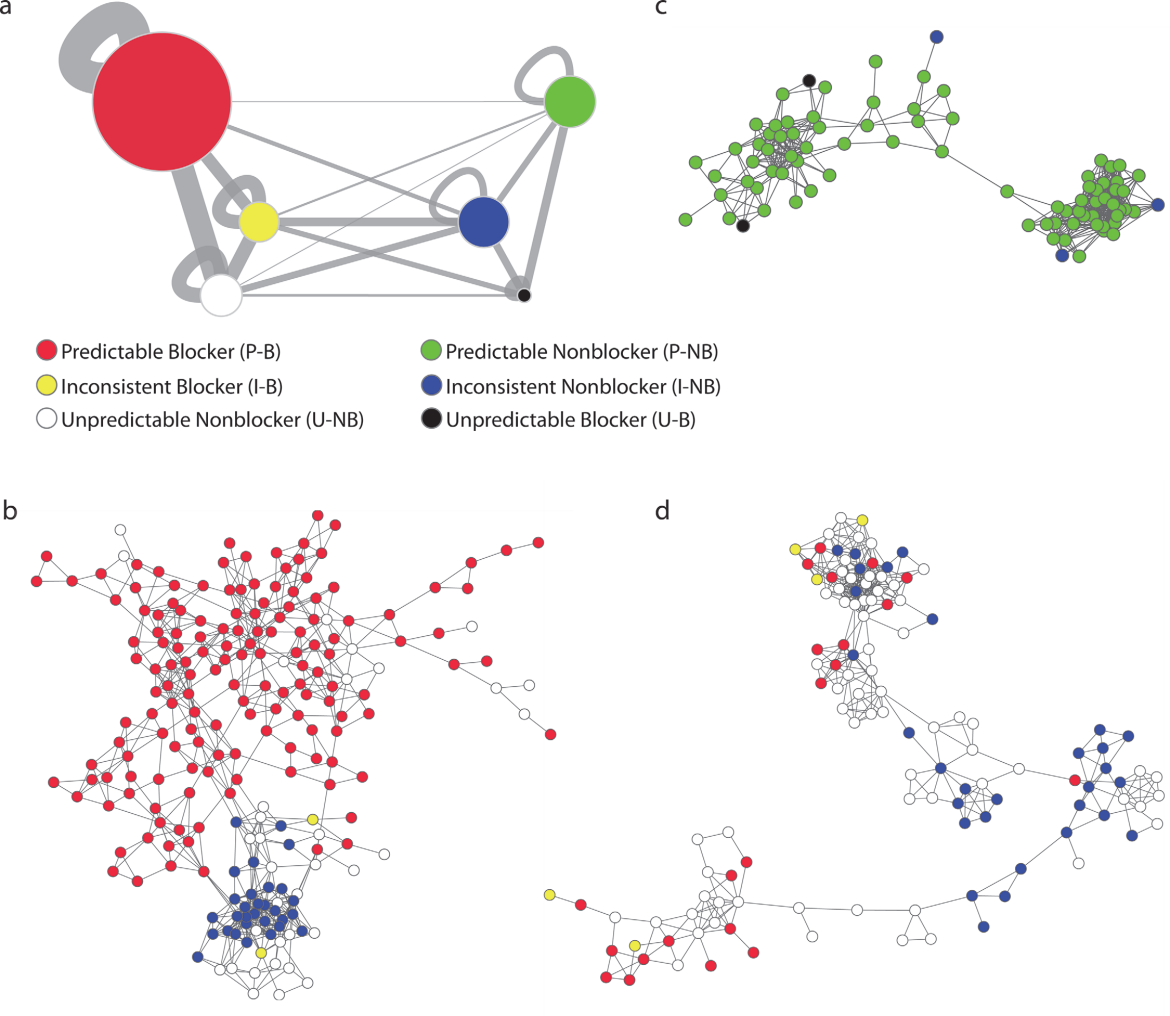
Structural similarity within and between six populations of compounds in the MLSMR assigned by activity-predictability classes. (**a**) Summary network visualizing the relationships of six possible combinations of activity (blocker and nonblocker) and predictability (predictable, inconsistent and unpredictable) classes. Each node represents the population of compounds of the same activity-predictability assignment, with edge width representing relative structural similarity quantified by relative connection density (see **Methods**), within and between each population using the structure network defined in **Fig 1**. Node sizes for P-B, I-B and U-B represent enrichment of blockers among compounds with high, intermediate, and low hBS compared to the distribution of the entire dataset. Similarly, P-NB, I-NB and U-NB sizes represent enrichment of nonblockers among compounds with low, intermediate, and high hBS. (**b**) An example cluster that highlights connection patterns within and between P-B, U-NB and I-NB compounds. (**c**) An example cluster that highlights connection patterns within and between P-NB, U-B and I-NB compounds. (**d**) An example cluster in which inconsistent and unpredictable compounds are more pronounced. Networks are generated using Cytoscape 2.8.2.

**Fig. 4A** is a summary network where nodes represent the population of compounds with a given activity-predictability class with edge width indicating relative structural similarity within and between each population. For the population of “predictable-blockers” (P-B) we observed pronounced structural self-similarity, and greater similarity to the “unpredictable-nonblockers” (U-NB) than “predictable nonblockers” (P-NB). **Fig. 4B** illustrates an example cluster of P-B compounds with limited connection to “inconsistent-nonblockers” (I-NB) but several intermingled U-NB compounds. Such islands represent regions of high-risk for hERG inhibition. Similarly, for compounds in the P-NB population (**Fig. 4A**), relatively higher similarity is observed within this group and with “unpredictable-blockers” (U-B) as well as “inconsistent-nonblockers” (I-NB). In contrast, little connectivity was observed to the P-B class as exemplified by the sample cluster in **Fig. 4C**, which constitutes a low-risk region for hERG liability. In comparison to compounds with extremely high and low hBS scores, the inconsistent classes (I-B and I-NB) demonstrate homogenous inter-class connectivity correlating with their poor *in silico* discrimination (**Fig. 4D**). Taken together, both the enrichment of true blockers among high hBS compounds and the relative structural similarities within and between the six predictability-activity classes suggest that the P-B population constitutes a high-risk space for hERG liability. Further, this analysis highlights regions of both tractable and ambiguous SAR with respect to hERG inhibition.

### Pharmacophore properties of predicted blockers

**Fig 5:**
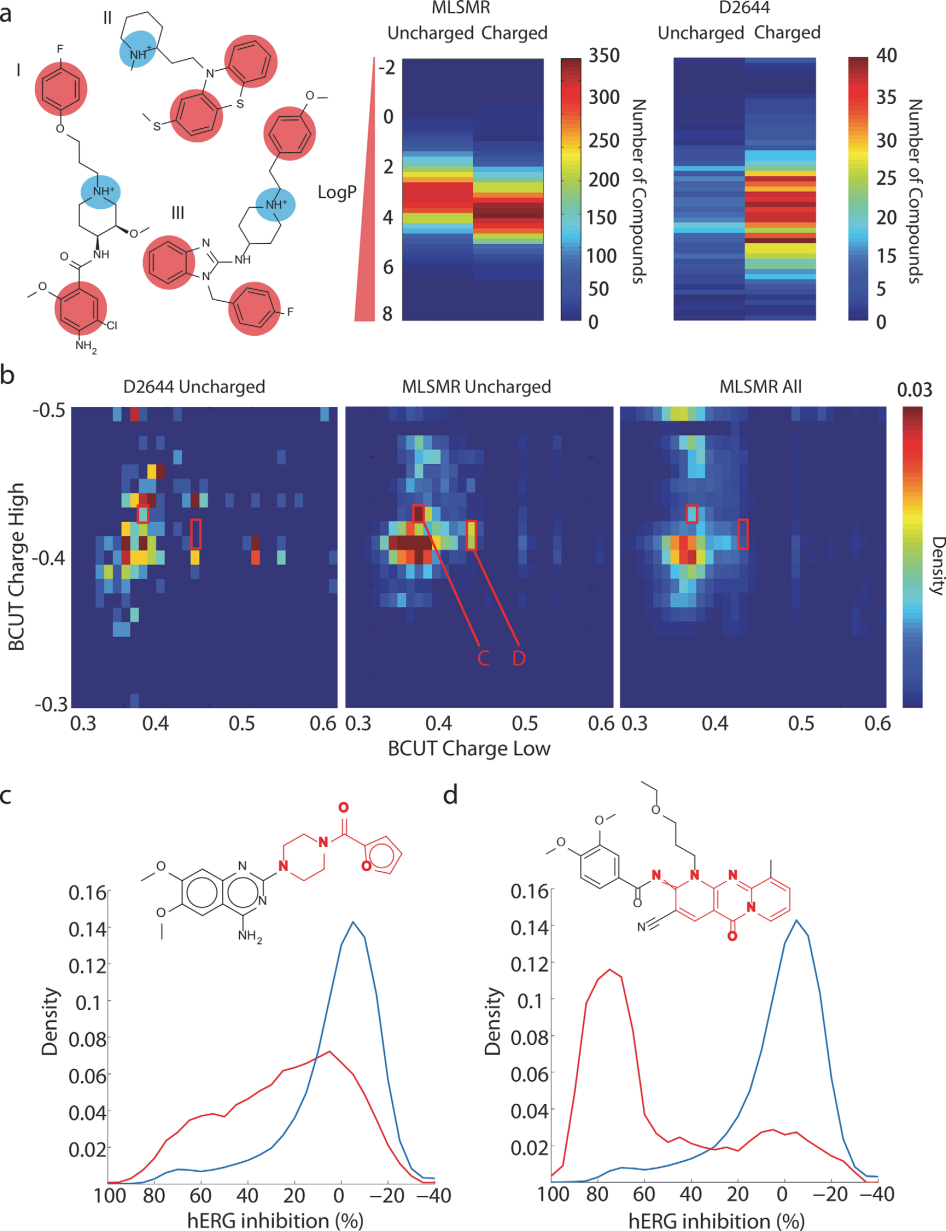
Novel structural determinants of hERG inhibitions. (**a**) (Left) Classical charged hERG pharmacophore consisting of positively charged basic nitrogen (blue) and hydrophobic groups (red), demonstrated by cisapride (I), thioridazine (II) and astemizole (III). (Right) Distribution and density of LogP values for neutral and charged hERG blockers in D2644 collection (right) and MLSMR (left). (**b**) Density of chemical space mapped using largest and smallest BCUT^TM^ charge descriptors for uncharged hERG blockers in D2644 (Left) and MLSMR (Center), and MLSMR library (Right). Red outlines denote enriched regions for neutral hERG blocker patterns in (**c**), (**d**). (**c**) Distribution of hERG inhibition for compounds containing prazosin fragment (red) compared to MLSMR library (blue). (**d**) As (**c**), for compounds containing illustrated triazatricyclo scaffold.

Earlier studies have identified several pharmacophores based on hERG blockers among known drugs [20–26,39], whose common features include charged basic nitrogens and hydrophobic groups that contribute to a large LogP value (**Fig. 5A**). Given that our studies now revealed a larger collection of hERG blockers, we examined whether and to what extent they exhibit these characteristic features. While a majority of the 1112 blockers in the D2644 collection of known drugs and hERG blockers contain this pattern, we find that in the MLSMR only about 50% of the predictable blockers (Fig. 4) are characterized by this charged motif (**Fig. 5A**). The novelty of these neutral blockers is emphasized by their poor prediction using models trained with the D2644 or D368 datasets (**S5A,B Fig.**). Furthermore, these neutral blockers exhibit different patterns than previously described neutral hERG pharmacophores [5], as none were detected as hits when these earlier pharmacophores were screened against the three dimensional conformers of our library available in PubChem3D [40]. Inspection of the chemical space covered by neutral MLSMR hERG blockers reveals regions not well-covered by the whole MLSMR library or neutral D2644 blockers (**Fig. 5B**). Examination of one of these clusters revealed many compounds containing a 1-(furan-2-carbonyl)piperazine moiety, whose inclusion in a molecule increases risk of hERG block > 10 μM by 4-fold (**Fig. 5C**). While this motif was previously observed only in the α-adrenergic antagonist prazosin [41], our analysis reveals this functional group in the context of multiple structures among MLSMR hERG blockers, suggesting that it may represent a previously unrecognized general modification that modularly increases hERG risk when added to a molecule. We also elucidated a tricylic scaffold which increases risk of hERG liability by 14-fold, and is unrepresented in any known blocker in the D2644 collection (**Fig. 5D**). Taken together, our results indicate both a higher prevalence of uncharged blockers violating the classical charged hERG pharmacophore pattern in the MLSMR versus known drugs, and reveal novel structural determinants of channel block derived from a modular segment of a known blocker and a completely novel scaffold. Representative electrophysiological traces for example compounds containing the patterns highlighted in Fig. 5 (C,D) are given in **S5 Fig.** Intriguingly, it appears that the prazosin moiety remains active when appended to compounds of different length (3 or 4 rings total), different terminal groups (Br and Cl versus a Nitro or Methyl) (**S5C Fig.**). The tricyclic scaffold appears more potent than the prazosin-fragment molecules at 1 μM concentration (**S5D Fig.**), suggesting that these core structures exhibit difference in intrinsic hERG inhibition potency that is not greatly influenced by substitutions on either core. These fragments are also larger than the maximal common substructures determined from analysis of the D2644 and D368 sets, which are primarily single rings with a short linker group [4,10]. PubChem identifiers for all neutral and charged blockers appear in **S1 Dataset**.

### External validation of ensemble modeling

**Fig 6:**
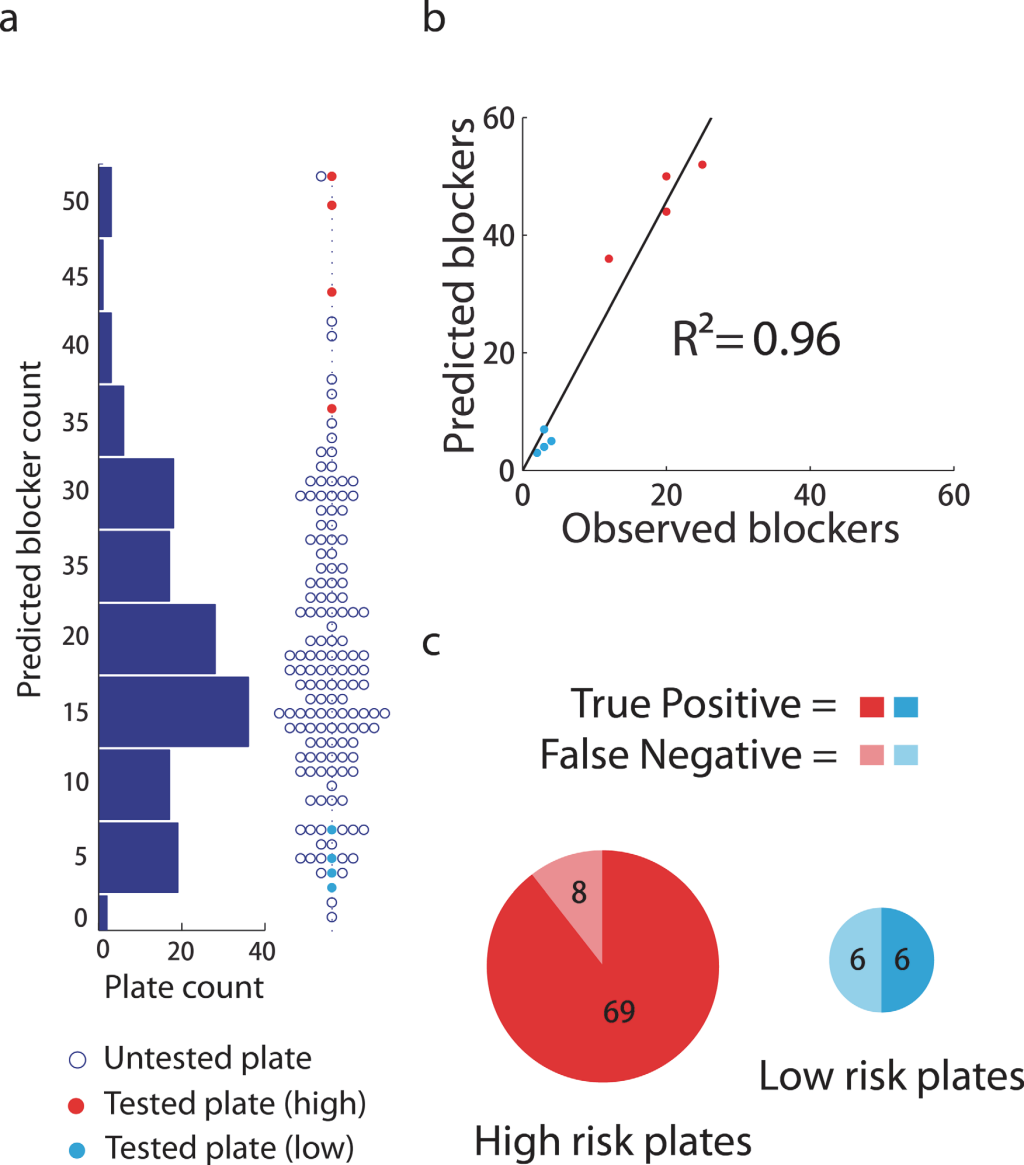
Experimental evaluation of MLSMR-derived ensemble hERG modeling. (**a**) Scatterplot and histogram distribution of predicted blocker numbers for ChemBridge DIVERSet 384-well plates. Experimentally evaluated plates for high (red) and low (light blue) predicted hERG inhibition are highlighted. (**b**) Correlation of predicted and experimentally observed blockers for eight test plates. (**c**) Pie graphs of true positive rate (recall) for high and low-risk plates at 10 μM concentration. Area is proportional to the number of experimentally observed blockers. Light color indicates false negatives, dark color true positives.

To evaluate whether our ensemble model based on our catalog of hERG-blocking chemical motifs could forecast population-level hERG liability in naïve compound populations, we generated an hBS profile for the 50,000 small molecules in the Chembridge DIVERSet. Plotting the results according to 384-well compound plate indicates a diversity of relative hERG risk judged by number of blockers (**Fig. 6A**). Based on the prediction, we selected eight plates representing high and low-risk samples for experimental evaluation. Following profiling, we calculated recall statistics of 90% and 50%, respectively, for experimentally determined blockers in the high and low-risk samples. These results validate that a majority of blockers were identified *in silico* by our methodology (**Fig. 6C**). A linear regression of the predicted on the observed results indicates an R^2^ of 0.96 (**Fig. 6B**). Furthermore, the experimental validation closely matches the predicted rank order of hERG liability for the eight plates (**Fig. 6A,B**). The fact that the number of predicted blockers for individual plates is systematically higher than observed indicates a possible bias in our predictions towards false positives.

The performance of individual compound predictions is shown in **S6A Fig.**, which illustrates receiver operating characteristic (ROC) curves for varying inhibition thresholds for classification. Because the active compounds represent 1%-6% of the overall data (dependent upon the inhibition threshold), the full ROC curves do not accurately represent the enrichment of inhibitors among the top of the ranked list of 50,000 compounds generated by the ensemble model. Thus, we have examined the partial ROC curves between 0–0.1 false positive rates, finding that the overall performance of the classification is similar in this region using multiple thresholds (**S6A Fig.**). However, like the MLSMR data, the predictive accuracy (true positive rate) is on average greatest for compounds with the highest potency, while moderate blockers exhibit higher misclassification rates (**S6 Fig.**). Furthermore, the variation of hBS for compounds over the full range of experimentally determined inhibition demonstrates that potent hERG blockers receive essentially uniform predictions (**S6B Fig.**), indicative of compounds occupying a high-risk region of chemical space. Taken together, these results suggest that structural neighborhoods revealed by analysis of the MLSMR data capture patterns present in naïve collections, and thus prospectively inform *in silico* diagnostics for chemical hERG liability.

## Discussion

Both the MLSMR and Chembridge DIVERSet validation dataset display correlation between the magnitude of hERG inhibition and consistency of *in silico* classification. Thus, our results suggest correlation between inhibitor potency and ‘smoothness’ of SAR in chemical clusters, a relationship highlighted by the neighborhood behavior of compounds in our network analyses and a qualitatively different property than that of previous predictive models. This pattern, illustrated by the ChC profile of Fig. 1, follows chemical intuition. The leftmost peak of the ChC curve represents molecular scaffolds such as illustrated in **Fig. 5D** with a high propensity for hERG liability. Conversely, greater structural heterogeneity among moderate inhibitors may reflect ‘dominant’ fragments that underlie hERG inhibition appended to a ‘recessive’ scaffold with many possible forms, such as the prazosin fragment highlighted in **Fig. 5C**. Thus, such analysis may allow dissection of chemical databases into both scaffolds and smaller fragments correlated with hERG liability or other biological endpoints.

Our analysis also revealed inactive molecules proximal to active neighborhoods, the ‘unpredictable’ compounds delineated by white nodes in Fig. 4. While the connections in our network do not explicitly represent the structural differences between adjacent compounds, previous work has sought to identify such side chains in large datasets [42,43]. Investigation of transformations characteristic of these ‘unpredictable’ compounds might reveal chemical groups that negate hERG inhibition, important information for therapeutic lead optimization. The mechanism of action of the newly identified blockers is not conclusively identified by our assay; while we note no major use-dependence in activity among these compounds (**S7 Fig.**), we cannot rule out reactions that might cause irreversible chemical modifications of the channel such as oxidation, which has previously been demonstrated to inhibit hERG current [44]. While this manuscript was under review, studies were published concerning hERG data for ~5,000 compounds in the ChEMBL database [45,46]. However, the data in this larger compound set compared to D2644 appears to confirm earlier hERG pharmacophore patterns of lipophilicity and basic nitrogen centers [45], without the novel scaffold patterns identified in our analysis of the MLSMR data.

In re-implementing previously described *in silico* hERG blocker classifiers, we converted continuous current inhibition measurement to binary categories using a 50% activity threshold. While this may induce unstable classification near the threshold and a continuous (regression) model can potentially perform better, our ensemble classifier nevertheless effectively ranks compound populations (e.g. 384-well plates) by relative hERG risk. Such methodology thus appears conducive to filtering libraries, allowing compound prioritization for a high-throughput campaign. Hence, our study represents several qualitative advances in hERG blocker prediction including (1) the necessity of including uncharged blockers for effective prediction of large collections (**S6 Fig.**), (2) a correlation between potency and *in silico* predictability (**Fig 3C, S6B Fig.**), and (3) effective population-based prediction of compound inhibition (**Fig. 6**). Taken together, these results advance our ability to computationally forecast hERG liability and define molecular populations amenable to such profiling.

## Methods

### High-throughput hERG profiling assay

The 318,496 unique members of the MLSMR library were diluted from 5 mM DMSO stock for high-throughput screening on the Ionworks Quattro (MDC, Sunnyvale, CA) in population patch clamp (PPC) mode (additional detail describing the library can be obtained from http://mlsmr.glpg.com). In PPC mode, compound effects are profiled in 384-well plates, with each well generating an averaged electrophysiological response from up to 64 individual cells upon compound treatment. The hERG profiling assay was performed as previously reported [13]. Briefly, Chinese hamster ovary (CHO) cells stably expressing the hERG channel were dislodged from tissue culture flasks and dispensed into PPC plates. Compound inhibition of hERG current density was then measured at 1 μM and 10 μM test concentrations. During the recording, background leak currents were estimated by a 100 ms step to -80 mV from an initial holding potential of -70 mV and subtracted from the subsequent current measurement. The campaign was conducted in two phases using slight differences in recording protocol. In the first phase, the initial voltage pulse used a 100 ms step to -30 mV from a holding potential of -70 mV, a 2 s conditioning step to +25 mV, and a 2 s test step to -30 mV. During the second phase of the screen, the potential during the conditioning step was raised to +45 mV. In both phases of the campaign, a secondary voltage pulse was applied using the same parameters as the second phase initial pulse. The initial and secondary pulses were applied in the absence of compound, and upon application of 1 μM and 10 μM test concentrations. Small molecule effects on hERG current density were quantified by measuring the peak tail current during the secondary voltage pulse prior to compound application and dividing by the amplitude following each test concentration. Recordings with peak tail current amplitude pre-compound > 0.2 nA, seal resistance > 30 MOhms, and seal resistance drop rate < 25% were retained for subsequent analysis. DMSO concentrations for diluted stock compounds were found to have no appreciable effect on hERG current density (data not shown).

### Datasets and descriptor calculation

Structural data for molecules annotated for hERG block used to develop binary classifiers by Robinson, et al. [10], and Doddareddy, et al. [4] were obtained from the original publications. The structure files for the MLSMR collection of 306,985 compounds screened in high-throughput format [37] were obtained from the PubChem database. Each collection was computationally filtered for solvent fragments, charge and stereochemistry standardized, and both canonical SMILES strings and molecular fingerprints were calculated using Pipeline Pilot v 6.1.5 (Scitegic, Inc. Accelrys). Additional molecular descriptors used to train the Winnow model and display the chemical space of uncharged hERG blockers were computed using the JChem (ChemAxon) *cxcalc* plugin.

### Pharmacophore analysis

Compound microspecies were set to the major microspecies at pH7.4, and the presence of charged ammine groups determined by a substructure search, both using JChem. Three dimensional distances for the six-point neutral hERG pharmacophore described in Aronov [5] were fit to three dimensional coordinates using multi-dimensional scaling. The six-point pharmacophore coordinates and all five-point pharmacophores generated by removing one of three hydrophobe features were screened against the all 515,478 three dimensional conformations of the MLSMR library available from PubChem3D [40] using the pharmer software [47].

### Chemical structure network analysis

We used three measures of structural similarity to define edges between the nodes (compounds) in the network formed from the MLSMR library.

The first is based on 2D compound-compound structural similarity, which we calculated using the Tanimoto coefficient (TC) between all pairs of compounds:

$$TC\left(i,j\right)\;=\;\frac{\left|F_{i}\cap F_{j}\right|}{\left|F_{i}\cup F_{j}\right|}$$

where *F*_*i*_, *F*_*j*_ are the set of bits present in fingerprints for compounds *i*, *j*. All pairwise similarity values were calculated in Pipeline Pilot using FCFP_6 fingerprints, and compounds were connected in this network if their similarity was greater than 0.7, a threshold previously used to compare chemical structure networks and *in silico* toxicology modeling [48,49].

The second class of network uses parent-child relationships among chemical scaffolds, with connections representing ring-group addition or removal from a structural core. For example, a two-ring parent scaffold would be linked by an edge to two ‘child’ scaffolds in this network, where each ‘child’ is generated by removing one of the core rings from the parent. The scaffold network was computed using the Java source code for Scaffold Hunter [28], modified to implement an exhaustive enumeration of all possible inter-scaffold connections [29].

Lastly, we computed a three-dimensional similarity network based on the conformer-based similars of each compound as calculated in PubChem3D using the OpenEye ROCS software [30]. Conformer-based similars in PubChem3D are defined as compounds with geometrically/volumetrically or pharmacophorically (arrangements of groups with donor/acceptor/ionic/hydrophobic properties) similar ‘shells’ in three dimensional space, using overlap measurement of these spatial components as the factors input to a Tanimoto calculation instead of structural fragments [50]. PubChem3D neighbors were downloaded from NCBI for each compound in the MLSMR library to construct this 3D similarity network.

To quantify the probability that compounds with similar hERG inhibition are adjacent in our structure network, we calculated a modified version of the rich-club coefficient. The rich-club coefficient is the ratio, for every degree *k*, of the number of actual to the number of potential edges for nodes with degree greater than *k* [31]. Here, instead of a degree cutoff, we threshold by percent inhibition to measure preferential similarity of potent blockers, a value we term the ‘chemical club coefficient’:

$$ChC\left(t\right)\;=\;\frac{2E_{t}}{\left(N_{t})(N_{t}-1\right)}$$

where *N*_*t*_ is the number of compounds/scaffolds exceeding a percentage inhibition threshold *t* and *E*_*t*_ is the number of structural connections between them. As a null model, this value is compared to *ChC (t)_rand_*, which is the same calculation performed for a network in which the activity annotations for all compounds have been randomly permuted.

The maximal ChC value is 1, indicating similarity among all hERG inhibitors greater than a given potency threshold *t*. To estimate the number of communities potentially represented by a smaller ChC number, consider the case of an even number of compounds which form discrete chemically similar pairs (2-cliques). This is the maximal number of communities for an even number of compounds, and for the next highest odd number. The number of edges between these compounds is then *N*_*t*_/2. Inserting this number into the formula for ChC yields and estimate of 1/ (*N*_*t*_-1) for the ChC of this example. This number may be compared to the empirical observation to determine if the number of communities is less than the upper bound of all 2-cliques. In our own data, the Log10(ChC) value of -3 for compounds with inhibition less than 86% is less than the values for maximal 2-cliques, which would be Log10(1/(197-1)) = -2.29. Thus, this number indicates that there are many singletons among these compounds, though the size of the communities formed by the connected compounds must be empirically examined.

### hERG binary classifier implementation

The Winnow linear classifier and SVM nonlinear classifier described in Robinson, et al. and Doddareddy, et al. were re-implemented using the original datasets. The Winnow model was implemented in C++ using as inputs ECFP_4 fingerprints and the discretized numerical descriptors, logP, maximum basic p*K*_a_, minimum acidic p*K*_a_ and Wiener Index. The SVM model was implemented using the MATLAB (MathWorks) libsvm toolbox [51], using 1024-bit FCFP_6 fingerprints as inputs and with model parameters (cost, *C* and kernel width, *γ*) optimized using a grid search and five-fold cross validation.

### Ensemble classification

The MLSMR collection was divided into five test folds. Compounds in each test fold were classified using a voting procedure trained on the remaining 80% of the data. To generate classifier votes, the training set for each test fold was divided into blocker and nonblocker categories. Winnow and SVM ensembles were then generated by training instances of each classifier on balanced samples including all test-set blockers and a random, equally sized set of nonblockers. This process was repeated *NB_MLSMR_/B_MLSMR_* times, where *NB_MLSMR_* and *B_MLSMR_* are the number of nonblockers and blockers in the training set. Thus, test-set compounds each received *NB_MLSMR_/B_MLSMR_* predictions. For the SVM model, optimal cost and kernel width parameters were re-used from the D2644 model and held constant for each balanced training batch in the majority voting procedure. The fraction of these predictions corresponding to the correct label was recorded as the predictability score (PS). PS values were discretized into three bins: predictable (P), for PS ≥ 0.95, inconsistent (I) for compounds with 0.95>PS>0.05, and unpredictable (U) for compounds with PS ≤ 0.05. Consensus classifications were generated by taking the intersection of classes P and U predicted by the Winnow and SVM models, with the remaining compounds assigned as I.

### Summary network calculation

The connection density *cd* for one group of compound nodes is defined by

$$cd\left(G\right)\;=\;\frac{E}{V(V-1)}$$

where *V* is the number of compounds in group *G*, and *E* is the number of edges between compounds in group *G*. The connection density between two groups *G*_1_ and *G*_2_ is calculated by

$$cd\left(G_{1},G_{2}\right)\;=\;\frac{E}{{V_{1}V}_{2}}$$

where *V*_1_, *V*_2_ are the number of compounds in groups *G*_1,_ *G*_2_ and *E* is the number of edges between compounds in the two groups.

The blocker/nonblocker or nonblocker/blocker ratios, as reflected by the node sizes in **Fig. 4A**, are used to measure the enrichment of blockers (nonblockers) in bins defined by our hERG blocker score (hBS) and predictability score (PS). Compounds in the MLSMR are classified into six bins according to hBS and PS: predictable-blockers (P-B), unpredictable-nonblockers (U-NB), inconsistent-blockers (I-B), inconsistent-nonblockers (I-NB), predictable-nonblockers (P-NB) and unpredictable-blockers (U-B). Denoting the number of blockers and nonblockers in the MLSMR as *B*_*MLSMR*_ and *NB*_*MLSMR*_, the enrichment of blockers for the P-B population, denoted by *E*_P-B_, is therefore

$$E_{P-B}\;=\;\frac{P\_B/(P\_B+U\_NB)}{B_{MLSMR}/(B_{MLSMR}+{NB}_{MLSMR})}$$

Similarly, the enrichments of blockers for the I-B and U-B populations are

$$E_{I-B}\;=\;\frac{\frac{I_{B}}{I_{B}+I_{NB}}}{\frac{B_{MLSMR}}{B_{MLSMR}+{NB}_{MLSMR}}}$$

$$E_{U-B}\;=\;\frac{U\_B/(U\_B+P\_NB)}{B_{MLSMR}/(B_{MLSMR}+{NB}_{MLSMR})}$$

The enrichment of nonblockers for the P-NB, I-NB and U-NB populations are

$$E_{P-NB}\;=\;\frac{P\_NB/(P\_NB+U\_B)}{{NB}_{MLSMR}/(B_{MLSMR}+{NB}_{MLSMR})}$$

$$E_{I-NB}\;=\;\frac{\frac{I_{NB}}{I_{NB}+I_{B}}}{\frac{{NB}_{MLSMR}}{B_{MLSMR}+{NB}_{MLSMR}}}$$

$$E_{U-NB}\;=\;\frac{U\_NB/(U\_NB+P\_B)}{{NB}_{MLSMR}/(B_{MLSMR}+{NB}_{MLSMR})}$$

### External Evaluation for Chembridge DIVERSet

The ensemble model derived from the MLSMR data was applied to the Chembridge DIVERSet collection, using a cutoff of 0.9 for average hBS-predicted blockers at 10 μM compound concentration by the two models. Four sample plates with a high and low predicted number of blockers were selected, with classification performance evaluated only for the 1,982 compounds in these plates not present in the MLSMR. The experimental evaluation used the second phase recording protocol described above, with compounds diluted from 10 mM DMSO stock.

## Supporting Information

S1 Dataset PubChem identifiers for all neutral and charged blockers. (XLSX)

S2 Dataset hERG inhibition for MLSMR dataset. (XLSX)

S3 Dataset PubChem identifiers (Substance IDs) for MLSMR dataset. (XLSX)

S1 Fig Chemical Club Coefficient distributions for scaffold and ROCS networks. (**a**) Scaffold Network Chemical-Club Coefficient distribution. (**b**) Same calculations as (**a**) performed for the MLSMR ROCS Network based on single-conformer 3D neighboring relationships. (**c**) Addition of ROCS neighboring relations to fingerprint-based ChC. (**d**) ChC following subtraction of known bioactives from the MLSMR library.
(TIF)

S2 Fig Distribution of physiochemical properties for MLSMR, D2644 and D368 datasets. (**a**) Molecular weight. (**b**) Octanol-water partition coefficient (ALogP). (**c**) Number of hydrogen bond donors. (**d**) Number of hydrogen bond acceptors. (**e**) Number of rotatable bonds. (**f**) Molecular polar surface area (PSA). Physicochemical properties were calculated using Pipeline Pilot 6.1.5 student edition.
(TIF)

S3 Fig Prediction results for Winnow and SVM models. Prediction performance is displayed as bar charts of the fraction of correctly and incorrectly predicted compounds in each bin of hERG inhibition: for example, the fraction of true positive (TP) and false negative (FN) in bins representing blockers, and fraction of true negative (TN) and false positive (FP) in bins representing nonblockers. True and false predictions are plotted on opposite sides of the horizontal line for visual clarity. Predictions of the test sets for the Winnow model by Robinson, et al., are shown in (**a-c**). (**a**) The model is trained and tested using the original published data set (D368). (**b**) The model is trained using the D368 dataset, and tested on the MLSMR dataset. (**c**) The model is trained with the MLSMR dataset and tested on the D368 dataset. Predictions of the test sets for the SVM model by Doddareddy, et al., are shown in (**d-f**). (**d**) The model is trained and tested using the original published data set (D2644). (**e**) The model is trained using the D2644 dataset, and tested on the MLSMR dataset. (**f**) The model is trained with the MLSMR dataset and tested on the D2644 dataset.
(TIF)

S4 Fig Prediction results for individual and combined hERG blockers models. Receiver-Operator Characteristic (ROC) curves for Winnow and SVM models, with partial Area Under the Curve (PAUC) calculated for false positive rate > 0.1.
(TIF)

S5 Fig Prediction results for Winnow and SVM models on neutral predictable blockers and representative traces for novel structural patterns among neutral hERG blockers in MLSMR. (**a**) Winnow model by Robinson, et al., trained with D368 dataset is used to predict neutral P-B compounds from **Fig. 5A**. (**b**) as in (**a**), for SVM model by Doddareddy, et al. trained with D2644 dataset. (**c**) Four neutral compounds with the fragment highlighted in **Fig. 5C** from the P-B population in **Fig. 4B**. (**d**) as in (**c**), for the scaffold highlighted in **Fig. 5D**.
(TIF)

S6 Fig Single-compound accuracy statistics for ensemble hERG classifier validation on Chembridge collection. (**a**) Receiver operating characteristic (ROC) plot of true positive rate (sensitivity) against false positive rate (1-specificity) for different classification thresholds for ensemble predictions of 1,982 Chembridge compounds (excluding duplicates of MLSMR compounds) in test plates for false positive rate < 0.1. For comparison the performance of a random classifier is indicated by a dashed diagonal line. (**b**) Distribution of prediction accuracy for compounds binned by experimental hERG inhibition at 10 μM concentration, plotted as fraction of true positive (TP) and false negative (FN) or true negative (TN) and false positive (FP) for compounds above (red) or below (light blue) the blocker threshold. Mean and standard deviation of hERG blocker score (hBS) is indicated by connected circles and error bars in each bin.
(TIF)

S7 Fig Activity-dependence of MLMSR hERG Inhibitors. The difference between hERG inhibition at 10 μM (vertical) is plotted versus the average inhibition of the two pulses (horizontal), with the no relationship trend line (red) and LOESS smoothed average (blue) indicated in overlay.
(TIF)

S1 Table Summary statistics of the D368, D2644 and MLSMR datasets. (DOCX)

S2 Table Prediction results for Winnow and SVM models with different datasets. (DOCX)
