# Supplementary figures and images for: Global Analysis Reveals Families of Chemical Motifs Enriched for hERG Inhibitors
Source: PLoS One. 2015 Feb 20;10(2):e0118324. doi: 10.1371/journal.pone.0118324 (PMC4336329; doi:10.1371/journal.pone.0118324)

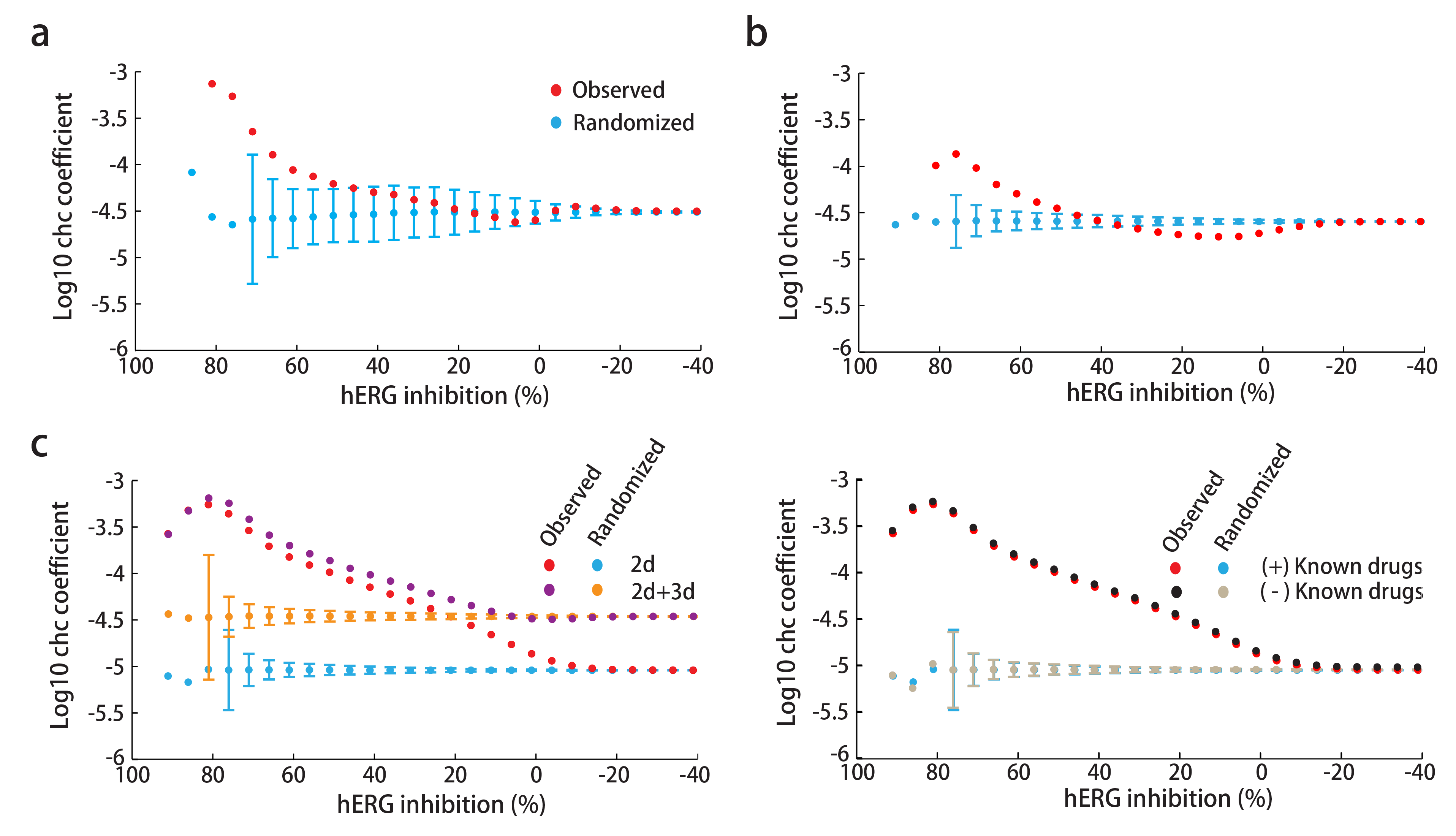

Supplement: S1 Fig — (a) Scaffold Network Chemical-Club Coefficient distribution. (b) Same calculations as (a) performed for the MLSMR ROCS Network based on single-conformer 3D neighboring relationships. (c) Addition of ROCS neighboring relations to fingerprint-based ChC. (d) ChC following subtraction of known bioactives from the MLSMR library. (TIF) [file pone.0118324.s004.tif]

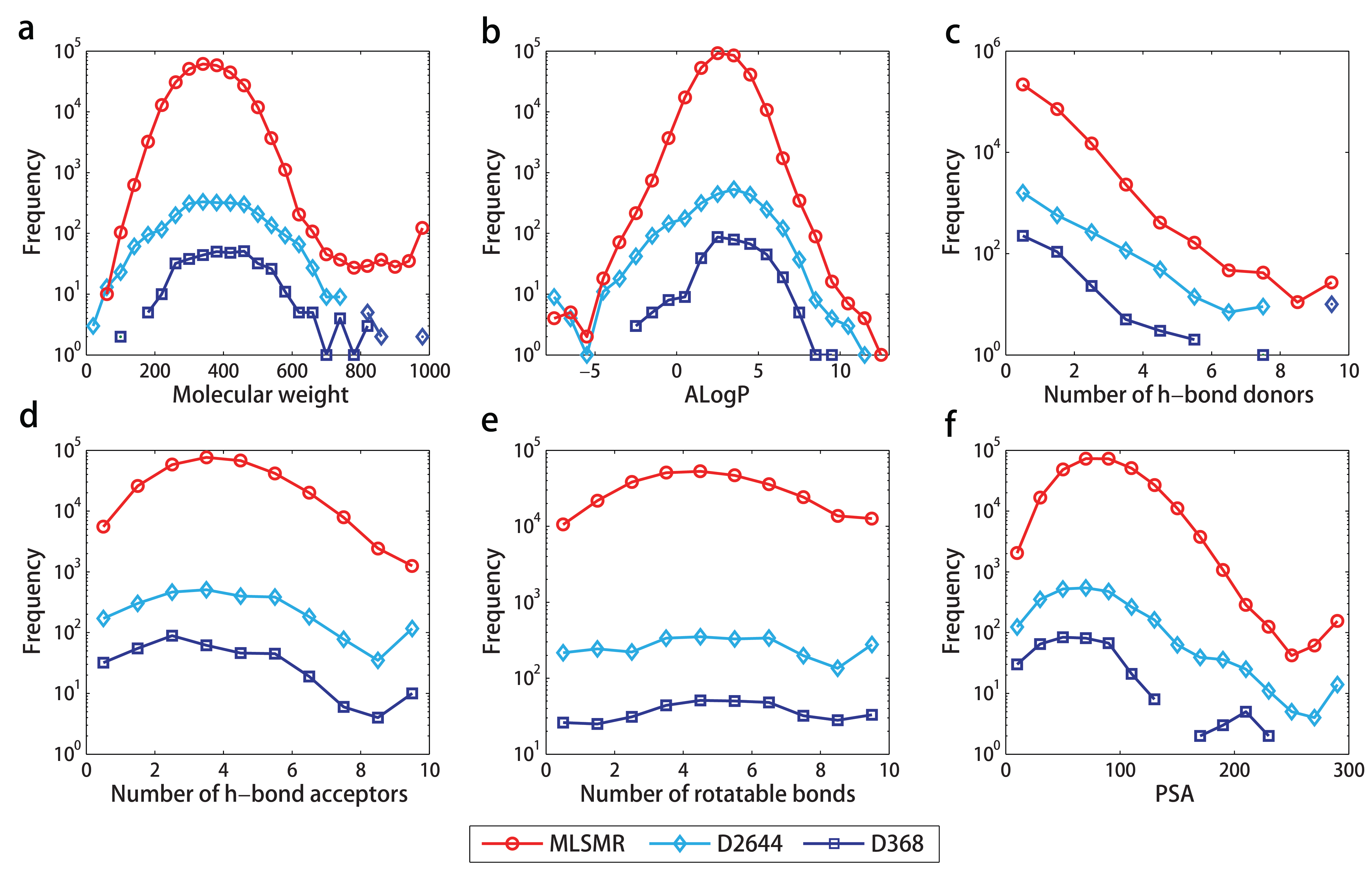

Supplement: S2 Fig — (a) Molecular weight. (b) Octanol-water partition coefficient (ALogP). (c) Number of hydrogen bond donors. (d) Number of hydrogen bond acceptors. (e) Number of rotatable bonds. (f) Molecular polar surface area (PSA). Physicochemical properties were calculated using Pipeline Pilot 6.1.5 student edition. (TIF) [file pone.0118324.s005.tif]

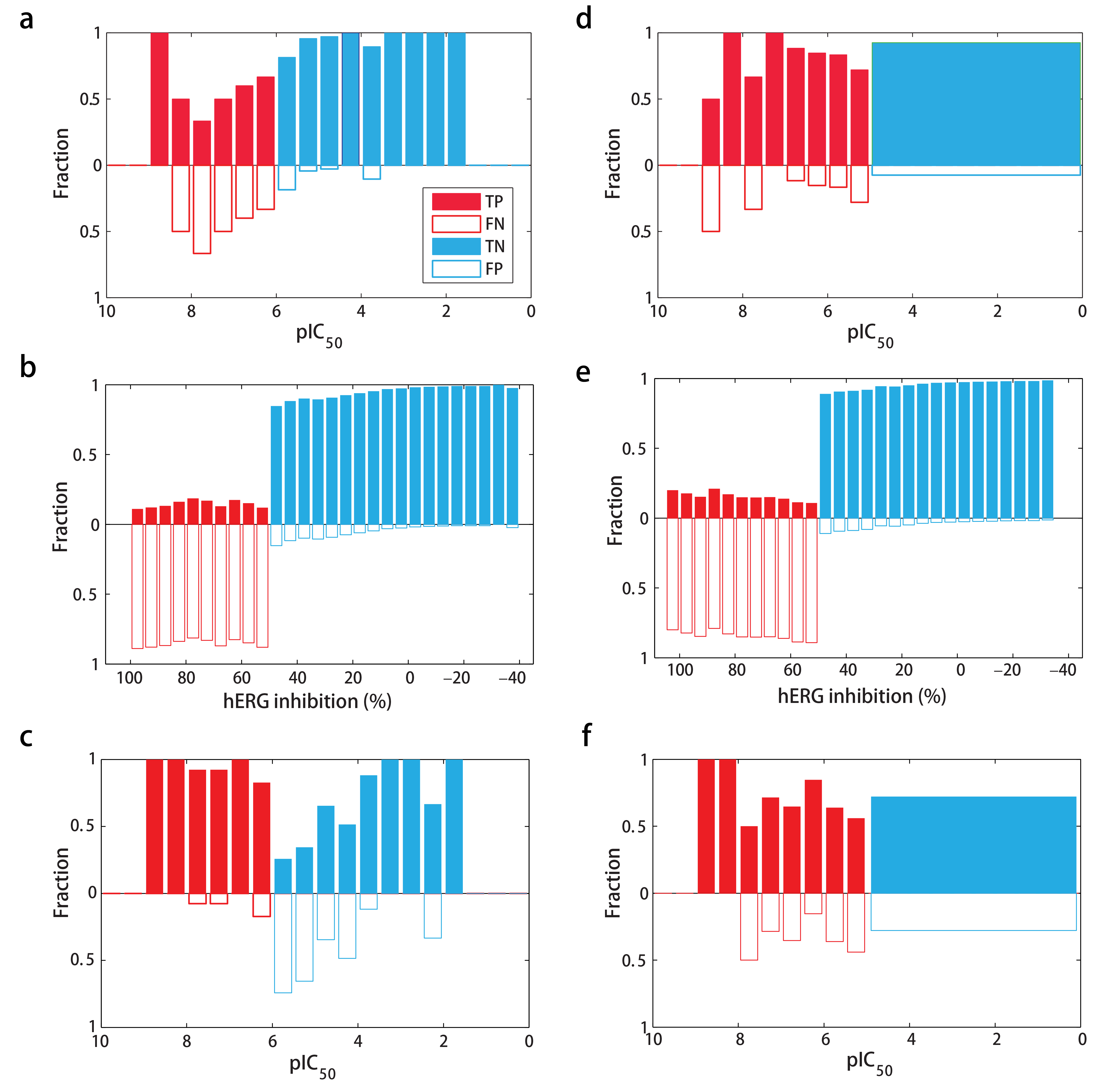

Supplement: S3 Fig — Prediction performance is displayed as bar charts of the fraction of correctly and incorrectly predicted compounds in each bin of hERG inhibition: for example, the fraction of true positive (TP) and false negative (FN) in bins representing blockers, and fraction of true negative (TN) and false positive (FP) in bins representing nonblockers. True and false predictions are plotted on opposite sides of the horizontal line for visual clarity. Predictions of the test sets for the Winnow model by Robinson, et al., are shown in (a-c). (a) The model is trained and tested using the original published data set (D368). (b) The model is trained using the D368 dataset, and tested on the MLSMR dataset. (c) The model is trained with the MLSMR dataset and tested on the D368 dataset. Predictions of the test sets for the SVM model by Doddareddy, et al., are shown in (d-f). (d) The model is trained and tested using the original published data set (D2644). (e) The model is trained using the D2644 dataset, and tested on the MLSMR dataset. (f) The model is trained with the MLSMR dataset and tested on the D2644 dataset. (TIF) [file pone.0118324.s006.tif]

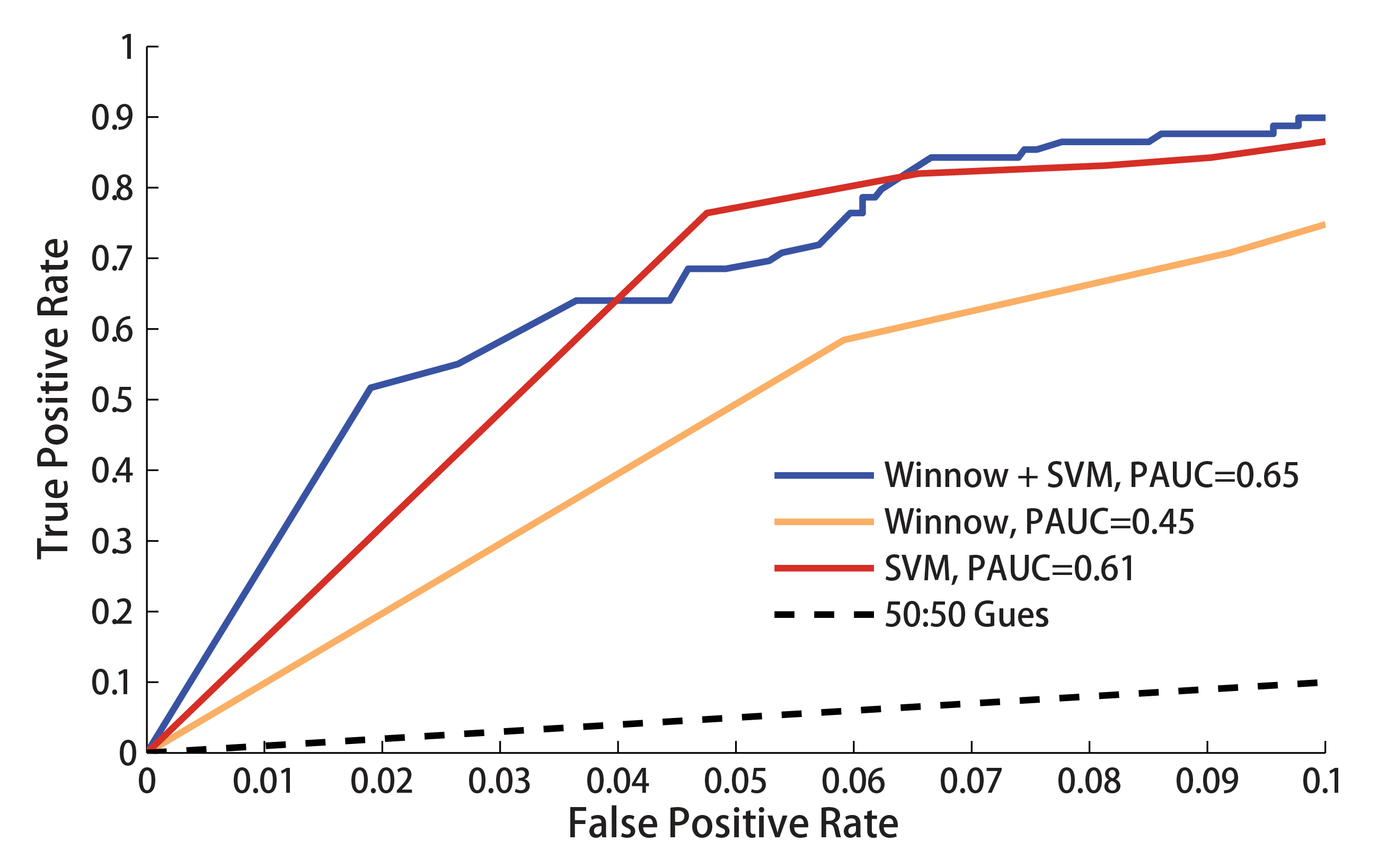

Supplement: S4 Fig — Receiver-Operator Characteristic (ROC) curves for Winnow and SVM models, with partial Area Under the Curve (PAUC) calculated for false positive rate > 0.1. (TIF) [file pone.0118324.s007.tif]

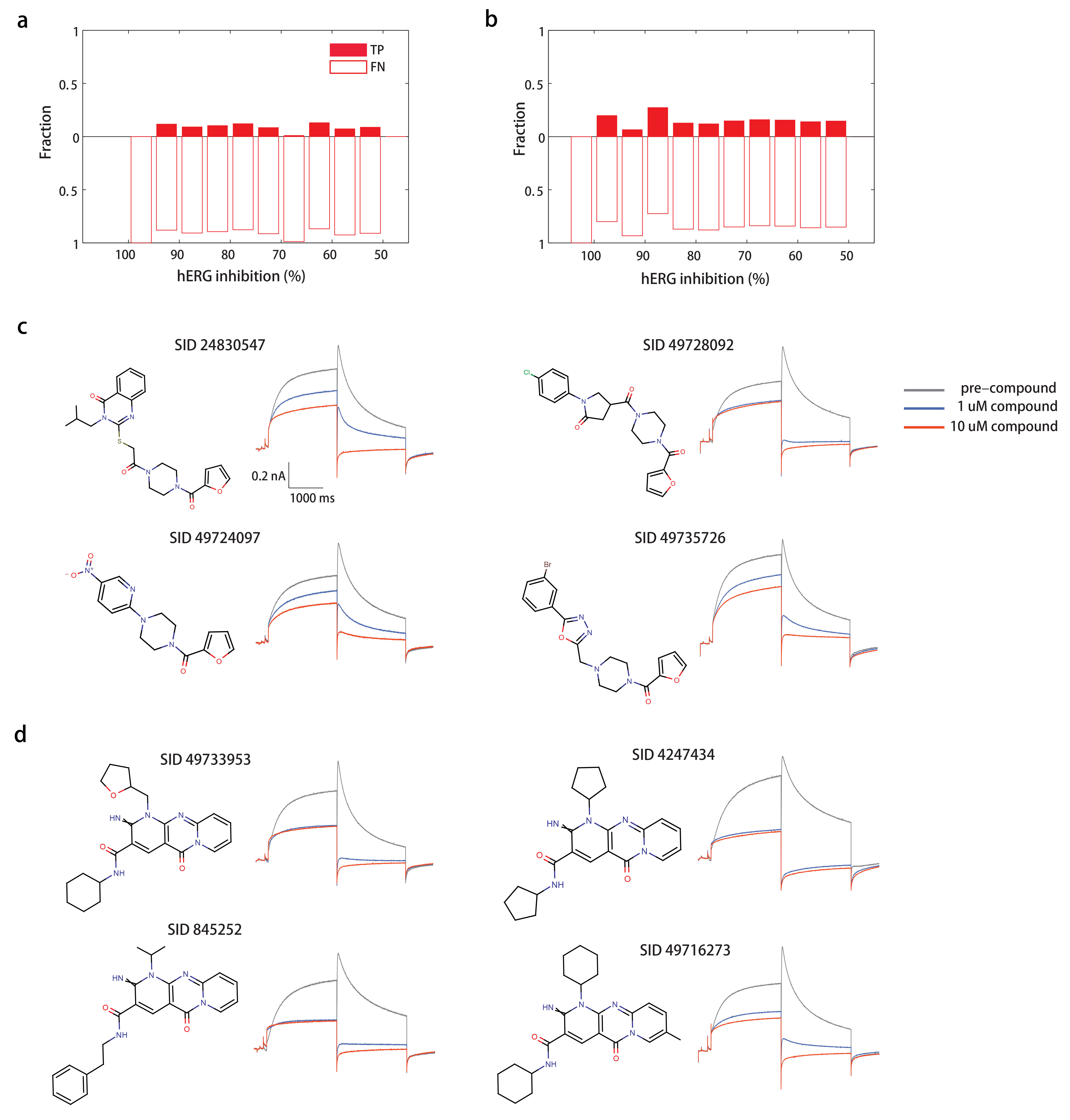

Supplement: S5 Fig — (a) Winnow model by Robinson, et al., trained with D368 dataset is used to predict neutral P-B compounds from Fig. 5A. (b) as in (a), for SVM model by Doddareddy, et al. trained with D2644 dataset. (c) Four neutral compounds with the fragment highlighted in Fig. 5C from the P-B population in Fig. 4B. (d) as in (c), for the scaffold highlighted in Fig. 5D. (TIF) [file pone.0118324.s008.tif]

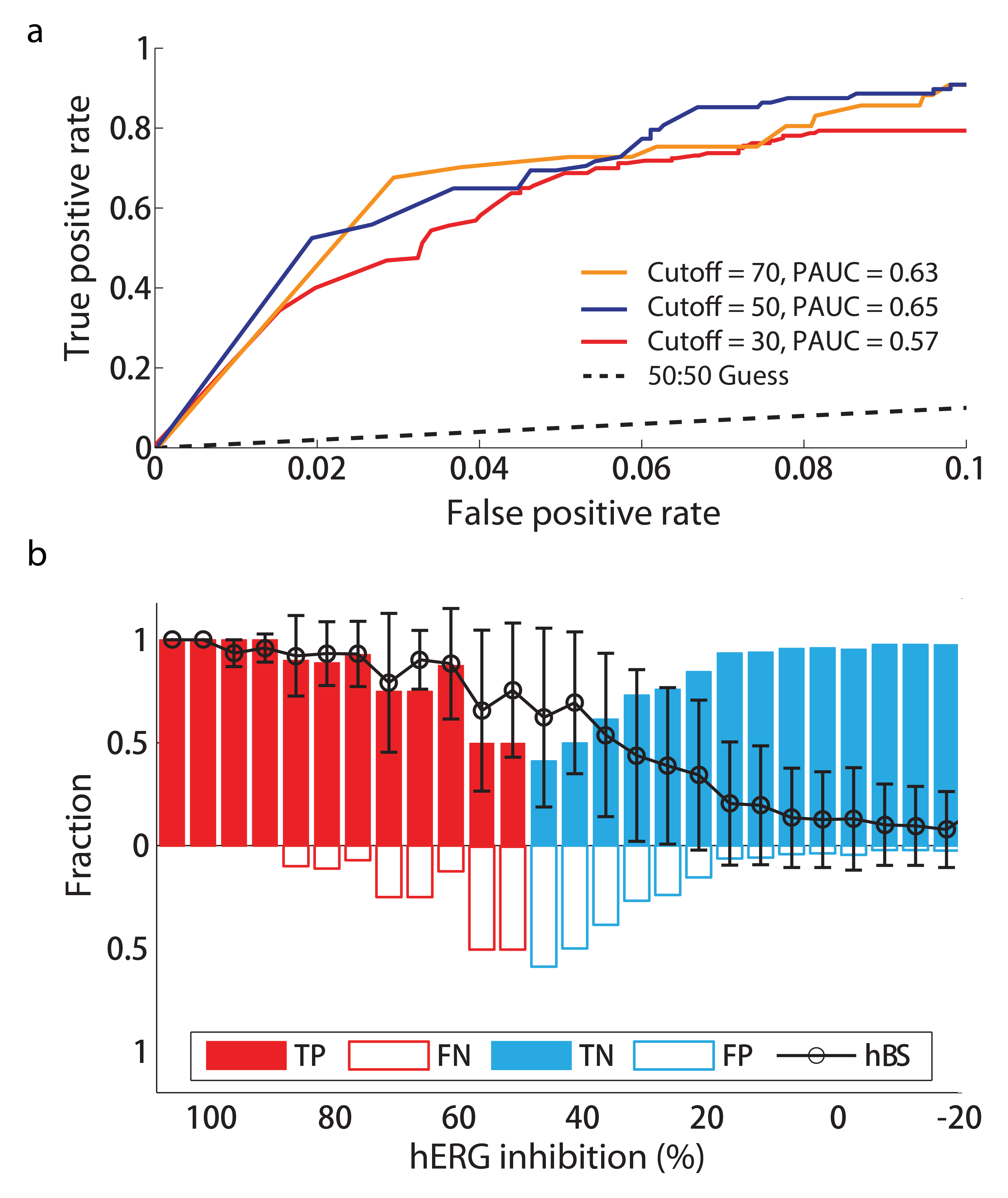

Supplement: S6 Fig — (a) Receiver operating characteristic (ROC) plot of true positive rate (sensitivity) against false positive rate (1-specificity) for different classification thresholds for ensemble predictions of 1,982 Chembridge compounds (excluding duplicates of MLSMR compounds) in test plates for false positive rate < 0.1. For comparison the performance of a random classifier is indicated by a dashed diagonal line. (b) Distribution of prediction accuracy for compounds binned by experimental hERG inhibition at 10 μM concentration, plotted as fraction of true positive (TP) and false negative (FN) or true negative (TN) and false positive (FP) for compounds above (red) or below (light blue) the blocker threshold. Mean and standard deviation of hERG blocker score (hBS) is indicated by connected circles and error bars in each bin. (TIF) [file pone.0118324.s009.tif]

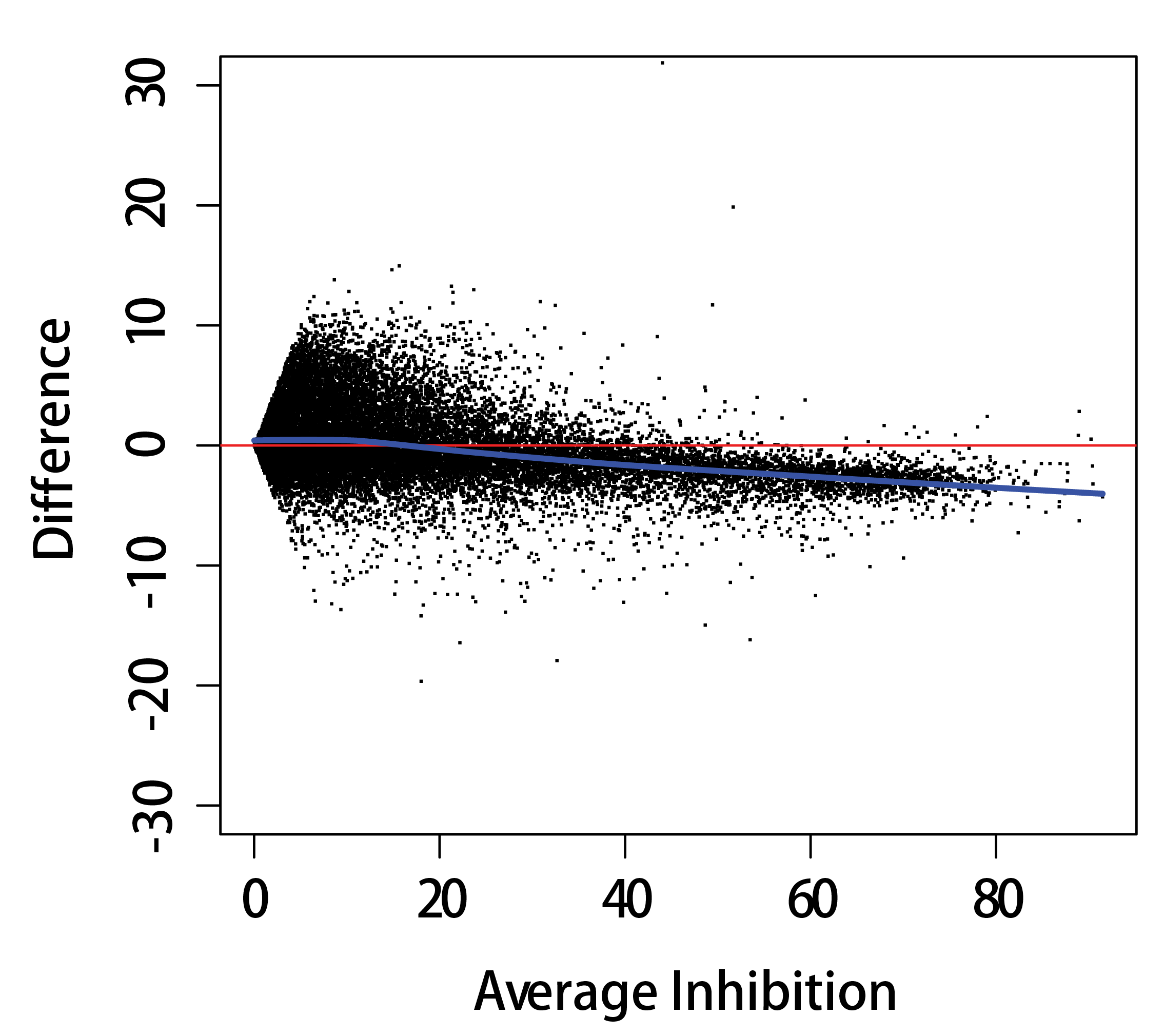

Supplement: S7 Fig — The difference between hERG inhibition at 10 μM (vertical) is plotted versus the average inhibition of the two pulses (horizontal), with the no relationship trend line (red) and LOESS smoothed average (blue) indicated in overlay. (TIF) [file pone.0118324.s010.tif]
