# Supplementary material for: Global Analysis Reveals Families of Chemical Motifs Enriched for hERG Inhibitors
Source: PLoS One. 2015 Feb 20;10(2):e0118324. doi: 10.1371/journal.pone.0118324 (PMC4336329; doi:10.1371/journal.pone.0118324)
Supplement: S1 Table — (DOCX) [file pone.0118324.s011.docx]

**S1 Table | Summary statistics of the D368, D2644 and MLSMR datasets**

| **Dataset** | **Size** | **Class Assignment** | | **Blockers** | **Nonblockers** | **Training Set (%)** |
| --- | --- | --- | --- | --- | --- | --- |
|  |  | **Blocker** | **Non-blocker** |  |  |  |
| D368 | 368 | $\text{IC}_{50}<1 \text{μM}$ | $\text{IC}_{50}\geq1 \text{μM}$ | 79 | 289 | 60 |
| D2644 | 2,644 | $\text{IC}_{50}<10 \text{μM}$ | $\text{IC}_{50}>30 \text{μM}$ | 1,112 | 1,432 | 90 |
| MLSMR | 306,895 | Inh.>50% (1 µM) | Inh.<50% (1 µM) | 2,219 | 304,676 | 80 |
|  |  | Inh.>50% (10 µM) | Inh.<50% (10 µM) | 13,744 | 293,151 | 80 |

In the MLSMR, thresholds of hERG current inhibition (Inh.) are used for the corresponding blocker/nonblocker class assignments as in the D368 and D2644 datasets.
