# Supplementary material for: Global Analysis Reveals Families of Chemical Motifs Enriched for hERG Inhibitors
Source: PLoS One. 2015 Feb 20;10(2):e0118324. doi: 10.1371/journal.pone.0118324 (PMC4336329; doi:10.1371/journal.pone.0118324)
Supplement: S2 Table — (DOCX) [file pone.0118324.s012.docx]

**S2 Table | Prediction results for Winnow and SVM models with different datasets**

| **Model** | **Training set** | **Test set** | **MCC** | **Acc** | **Rec (B)** | **Prec (B)** | **Rec (NB)** | **Prec (NB)** |
| --- | --- | --- | --- | --- | --- | --- | --- | --- |
| Winnow | D368 training set | D368 test set | 0.51 | 0.87 | 0.55 | 0.65 | 0.93 | 0.92 |
| Winnow | D368 training set | MLSMR | 0.25 | 0.85 | 0.15 | 0.61 | 0.98 | 0.86 |
| Winnow | MLSMR training set | D368 test set | 0.37 | 0.62 | 0.91 | 0.35 | 0.54 | 0.96 |
| SVM | D2644 training set | D2644 test set | 0.75 | 0.88 | 0.81 | 0.89 | 0.93 | 0.87 |
| SVM | D2644 training set | MLSMR | 0.19 | 0.62 | 0.14 | 0.76 | 0.97 | 0.60 |
| SVM | MLSMR training set | D2644 test set | 0.40 | 0.71 | 0.56 | 0.70 | 0.82 | 0.72 |

MCC denotes Matthews Correlation Coefficient; Recall (Rec) is the fraction of instances in a class (blocker, B or nonblocker, NB) correctly assigned to the class; Precision (Prec) is the fraction of instances assigned to a class that belong to that class; Accuracy (Acc) is the fraction of overall correct predictions. For a better comparison, the MCC for the Winnow model applied to the MLSMR data is calculated so that the blocker/nonblocker ratio in the MLSMR is the same as that in the D368 test set (24 blockers and 124 nonblockers). This is achieved by drawing 1000 subsets of MLSMR predictions, each containing predictions of all 2,219 blocker compounds and predictions of a subset of nonblocker compounds (here 11,465 nonblockers to keep the same ratio as in D368) randomly sampled from all 304,676 nonblocker compounds in MLSMR. The overall MCC is the arithmetic mean of 1000 MCCs obtained in this way. The MCC calculation for the SVM model is obtained in the same way but using a blocker/nonblocker ratio as in D2644 (108 blockers and 147 nonblockers).
